# Secreted Protein Acidic and Rich in Cysteine (SPARC) Exacerbates Colonic Inflammatory Symptoms in Dextran Sodium Sulphate-Induced Murine Colitis

**DOI:** 10.1371/journal.pone.0077575

**Published:** 2013-10-21

**Authors:** Yoke-Leng Ng, Borut Klopcic, Frances Lloyd, Cynthia Forrest, Wayne Greene, Ian C. Lawrance

**Affiliations:** 1 Centre for Inflammatory Bowel Diseases, School of Medicine and Pharmacology, University of Western Australia, Fremantle, Western Australia, Australia; 2 School of Veterinary and Biomedical Sciences, Murdoch University, Perth, Western Australia, Australia; 3 School of Pathology and Laboratory Medicine, University of Western Australia, Fremantle, Western Australia, Australia; Virginia Tech, United States of America

## Abstract

**Background:**

Secreted Protein Acidic and Rich in Cysteine (SPARC) is expressed during tissue repair and regulates cellular proliferation, migration and cytokine expression. The aim was to determine if SPARC modifies intestinal inflammation.

**Methods:**

Wild-type (WT) and SPARC-null (KO) mice received 3% dextran sodium sulphate (DSS) for 7 days. Inflammation was assessed endoscopically, clinically and histologically. IL-1β, IL-4, IL-5, IL-6, IL-10, IL-13, IL-17A, IL-12/IL23p40, TNF-α, IFN-γ, RANTES, MCP-1, MIP-1α, MIP-1β, MIG and TGF-β1 levels were measured by ELISA and cytometric bead array. Inflammatory cells were characterised by CD68, Ly6G, F4/80 and CD11b immunofluorescence staining and regulatory T cells from spleen and mesenteric lymph nodes were assessed by flow cytometry.

**Results:**

KO mice had less weight loss and diarrhoea with less endoscopic and histological inflammation than WT animals. By day 35, all (n = 13) KO animals completely resolved the inflammation compared to 7 of 14 WT mice (p<0.01). Compared to WTs, KO animals at day 7 had less IL1β (p = 0.025) and MIG (p = 0.031) with higher TGFβ1 (p = 0.017) expression and a greater percentage of FoxP3+ regulatory T cells in the spleen and draining lymph nodes of KO animals (p<0.01). KO mice also had fewer CD68+ and F4/80+ macrophages, Ly6G+ neutrophils and CD11b+ cells infiltrating the inflamed colon.

**Conclusions:**

Compared to WT, SPARC KO mice had less inflammation with fewer inflammatory cells and more regulatory T cells. Together, with increased TGF-β1 levels, this could aid in the more rapid resolution of inflammation and restoration of the intestinal mucosa suggesting that the presence of SPARC increases intestinal inflammation.

## Introduction

The Inflammatory Bowel diseases (IBDs), Crohn’s disease and ulcerative colitis are chronic inflammatory disorders that affect the human gastrointestinal tract. Numerous murine models have been developed to investigate the pathology that underlies IBD inflammation and the dextran sodium sulphate (DSS)-induced model of colitis is one of the most widely used. DSS is directly toxic to gut epithelial cells located within basal crypts, and reduces mucosal barrier integrity allowing the translocation of antigens from the intestinal lumen into the submucosa. The presence of luminal antigens in the intestinal mucosa can then promote acute inflammation with recruitment, and activation, of immune cells [1], [2]. While the destructive effects of DSS are generally reversible upon DSS withdrawal [3], the administration of DSS to mice over an extended period of time can induce chronic intestinal inflammation with symptoms and histological features similar to those observed in human IBD [1], [4], [5].

Secreted protein acidic and rich in cysteine (SPARC) is a matricellular glycoprotein involved in diverse biological processes, including tissue remodelling, wound repair, angiogenesis as well as cellular differentiation, adhesion, proliferation and migration [6]–[8]. A deficiency in SPARC has been associated with a reduction in both renal inflammation and fibrosis [9], but these effects may not be universal across different tissues as demonstrated by the bleomycin-induced lung injury in SPARC knock out (KO) mice. This model demonstrated that in SPARC KO animals, there was increased neutrophil recruitment to the sites of acute inflammation with enhanced levels of inflammation, but not necessarily with the development of fibrosis [10].

The pro-inflammatory cytokines such as interleukin (IL)-1β, IL-6 and tumour necrosis factor (TNF)-α promote inflammation, which is central to leucocyte recruitment and activation with the secretion of various growth factors including platelet derived growth factor and transforming growth factor (TGF)-β. SPARC has been suggested to modulate inflammation through the regulation of cytokine and chemokine bioavailability through its effects on the production and activity of metalloproteinases (MMPs) such as MMP-2 and MMP-3 [11], [12]. The increase in these MMPs enhances the cleavage of pro-IL1β to active IL-1β [13] and thus the development of inflammation [14], [15].

Through endoscopic, clinical and histological evaluation, together with the determination of pro-inflammatory cytokines levels within the colonic tissue, this study aimed to investigate the contribution that SPARC may have on the development of intestinal inflammation. SPARC KO and wild type (WT) mice received DSS to induce an acute colitis and were assessed over time for tissue reconstitution. The findings demonstrated that SPARC KO mice had significantly less inflammation, including lower endoscopic and histology scores with more regulatory T (Treg) cells, and less inflammatory cells in combination with lower pro-inflammatory cytokine levels and a possible faster healing rate. These findings suggest that SPARC is able to enhance inflammation in the DSS induced murine model of colitis.

## Materials and Methods

### Ethics Statement

The care and use of the animals and experimental protocol were approved by the Animal Ethics Committee (AEC) of the University of Western Australia (Animal ethics number: RA/3/100/606).

129/SvJ × C57/BL6 WT and SPARC KO mice were obtained as a gift from Dr E. Helene. Sage, Hope Heart Institute, Washington State, USA on a mixed background [16]. Animals were maintained under specific pathogen free conditions. Unless specifically stated, female mice aged 10–12 weeks were used in all experiments. Only female mice were used in order to reduce potential variability between the findings, ease of use and the lack of aggressive behavior that necessitates the male mice being caged separately. Mice were allowed food and water *al libitum*. Confirmation of complete ablation of SPARC gene was demonstrated in all SPARC null mice by genotyping using PCR.

### DSS induction of colitis

**Figure 1:**
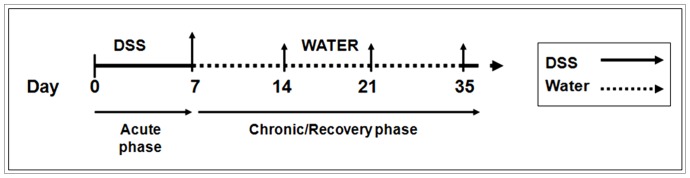
Overview of the experimental design to investigate acute and recovery phase. Mice receiving DSS for 7 days were used to examine the acute inflammatory response. Mice that received 3% DSS for 7 days followed by plain tap water for 1, 2 and 4 weeks (D14, D21 and D35 respectively) were used to investigate the recovery. Age matched control groups (non DSS treated) were present for each time point. (↑ represents points of analysis).

Colitis was induced by the addition of DSS [3% (wt/vol) (MP Biomedicals LLC, Aus)] to the drinking tap water for 7 days. Control mice received plain tap water without DSS. As the efficacy of DSS varies between batches, the majority of the experiments were undertaken using the same batch number. Each new DSS batch was tested for efficacy. Following induction there was a recovery period of 1–7 weeks without DSS (***Figure 1***). The mean daily DSS-water consumption was recorded for each group. Mice were assessed daily during the treatment period with body weights and stool samples examined, and weekly during the recovery period.

### Endoscopic monitoring of colitis

A high resolution mouse video endoscopic system was used to assess the level of colitis and the mice were routinely scoped 1 day prior to sacrifice. Mice were anaesthetised using an intraperitoneal ketamine/xylazil injection of a 0.01 ml/g body weight. The experimental endoscopy setup, denoted "*Coloview system*", consisted of a miniature endoscope (scope 1.9 mm outer diameter), a xenon light source, a triple chip camera, and an air pump (Karl Storz, Germany) to achieve regulated inflation of the mouse colon. The endoscopic procedure was viewed on a colour monitor and digitally recorded. All endoscopic procedures were blindly assessed.

The level of colitis was determined using the modified murine endoscopic score of colitis severity (MEICS) [17]. The MEICS system consists of 5 parameters: Thickening of the colon wall, changes of the normal vascular pattern, presence of fibrin, mucosal granularity and stool consistency. Endoscopic grading was performed for each parameter (scored between 0 and 3) leading to a cumulative score of between 0 (no signs of inflammation) and 15 (endoscopic signs of very severe inflammation). Healthy mice usually have a score of 0–3.

### Tissue dissection

The colon was removed and the length measured from the anus to caecum. Approximately 1 mm from the anus was discarded to remove the rectum as it has a different tissue fibro-structure to the colon. The spleen and mesenteric lymph nodes were removed stored in supplemented DMEM (Gibco® Invitrogen Co., Aus) and kept on ice until use. Colonic tissue for cytokine detection was washed in RPMI-1640 (Gibco® Invitrogen Co., Aus) supplemented with antibiotics. The tissues were placed in 96 well trays containing serum free RPMI-1640 supplemented with antibiotics for 24 hours. The culture supernatants were collected, spun at 12000 g at 4°C, snap frozen and stored at –80°C until use.

### Preparation of paraffin sections

Colonic tissue was cleaned, formalin-fixed and embedded in paraffin wax. Sections, 4.5μm thick were dried at 55°C overnight and stored at room temperature (RT) until stained with haemotocylin and eosin (H&E) using the Shandon Linistain™ GLX Linear Stainer (Thermo Scientific, AUS).

All H&E stained paraffin sections were assessed blindly by specialist gastroenterological histopathologist according to the scoring system by Dieleman *et al* [18]. In this scoring system, 4 features are graded**:** The severity and depth of inflammation are each scored from 0–3, while the level of crypt damage and regeneration are each scored between 0 and 4. These changes were also quantified as to the percentage of the total colonic circumference that was involvement by the disease process: i) 1–25%; ii) 26–50%; iii) 51–75%; iv) 76–100%. A histological score (between 0–56) was calculated for each sample based on multiplying the score of each feature by the percentage of the tissue involved and adding the totals together. At the day 35 time-point, the inflammation severity and regeneration scores were added and compared using chi square by SPSS (Version 19, IBM, USA).

### Immunofluorescent staining of cryosections

Colons were embedded in Tissue-Tek® O.C.T freezing medium (ProSciTech, Aus), snap frozen in liquid nitrogen and stored at –80°C. The tissues were cut into 7μm thick sections on a freezing microtome cryostat chamber and sections mounted onto Superfrost™ slides, dried at RT and stored at –80°C. Prior to use, slides were fixed in ice cold acetone for 2 minutes, rehydrated in PBS and blocked with 5% goat serum. The sections were incubated in the optimal concentration of rat monoclonal primary antibody (**CD68**, Kp1, 1:400, abcam, USA; **Ly6G** 1A8, 1:400, Biolegend, USA; **F4/80** CI:A3-1, 1:200, AbD serotec, USA; **CD 11b**, M1/70, 1:400, BD Pharmingen, USA) prepared in commercial antibody diluent (DAKO, Denmark) overnight at 4°C and then washed 3 times for 10 minutes in PBS. The sections were then incubated in goat anti-rat IgG secondary antibody conjugated to Alexa Fluor® 594 (Invitrogen Co., USA) for 1 hour at RT in a dark, humidified chamber. After washing a further 3 times in PBS, slides were mounted with Prolong Gold Antifade reagent with DAPI (Invitrogen Co., Aus). Slides were viewed and photographed with Olympus IX-81 inverted fluorescence microscope. The DAPI stained nuclei blue when viewed under the ultra violet (UV) filter while the proteins of interest stained red when view with green filter. Each section was viewed at x200 magnification and photographed. The numbers of positive cells were counted in 5–15 fields.

### Detection of cytokines

A customized 15plex BD™ Cytometric bead array (CBA) Flex set was used to detect the following cytokines and chemokines simultaneously in the tissue explants and serum samples from individual mice: IL-1β, IL-4, IL-5, IL-6, IL-10, IL-13, IL-17A, IL-12/IL23p40, TNF-α, IFN-γ; RANTES, MCP-1, MIP-1α, MIP-1β, MIG. Supernatants from tissue explants were used undiluted (except 1:60 for IL-6 analysis). The assay was set up in multiscreen HTS filter plates (Milipore, USA) and performed according to the manufacturer’s instructions. A typical standard was obtained by serial dilution of recombinant protein. Samples were analysed on a BD FACSCanto™ instrument using BD FACSDIVA™ software and BD FCAP Array™ software. All cytokines were measured at the protein level without *ex vivo* stimulation of the cells/tissues (serum free) as this may reflect the *in vivo* conditions better [5].

To measure TGF-β protein levels the Quantikine® mouse/rat/porcine/canine immunoassy for mouse TGF-β (R&D Systems, Aus) was used according to the manufacturer’s instructions modified to include neutralisation of the sample with 2.4 N NaOH instead of 1.2N NaOH following activation with acid. The explant culture supernatants were diluted 1.3 fold following sample activation. Recombinant protein from 31.2 to 2000 pg/ml served as a standard curve.

### Flow cytometry analysis

Single cell suspensions from the spleen and mesenteric lymph nodes (MLN) were filtered through a 40μm wire mesh and the red blood cells were lysed with red cell lysis buffer. The single cells were suspended in supplemented DMEM at 4°C till staining. All staining for Treg cells was performed using the mouse regulatory T cell staining kit (eBioscience, USA) according to the manufacturer’s instructions.

All data were collected with BD FACSCanto™ instrument using BD FACSDIVA™ software with analysis by FlowJo software (version 7.6, Tree Star Inc, USA). The lymphocyte population was identified on the basis of size and granularity. The lymphocytes were further characterized by their cell surface markers. All gating was set based on the appropriate isotype control. In order to quantify the regulatory T cells, the CD4+ CD25+ population within the lymphocyte population was selected and further gating with FoxP3 was used to analyse Treg cells. For each sample 100 000 gated lymphocyte events were recorded. The results represent the pooled data of mean percentage of cells of interest out of the total gated cells from 1–3 independent experiments.

### Statistical Analysis

Statistical significance was calculated using GraphPad Prism (Version 5.0) (GraphPad software, Inc, USA). All graphs and comparison of differences between groups were assessed using Student’s unpaired t-test unless otherwise mentioned. The data from the cell counting of the immunofluorescence sections were sampled from a Poisson distribution, the analysis applied was loglinear regression, and the differences between WT and SPARC KO mice were compared using a logliner regression analysis by SPSS (Version 19, IBM, USA).

## Results

### Clinical symptoms, endoscopic and histopathalogical inflammation

All DSS-treated mice demonstrated body weight loss and bloody liquid stools 2–7 days after commencing DSS. The WT mice had a greater percentage of weight loss than SPARC KO mice although this was not statistically significant (WT: 28.09% vs SPARC KO: 12.36%, p = 0.08). Both groups, however, regained the lost weight once DSS treatment was ceased.

**Figure 2:**
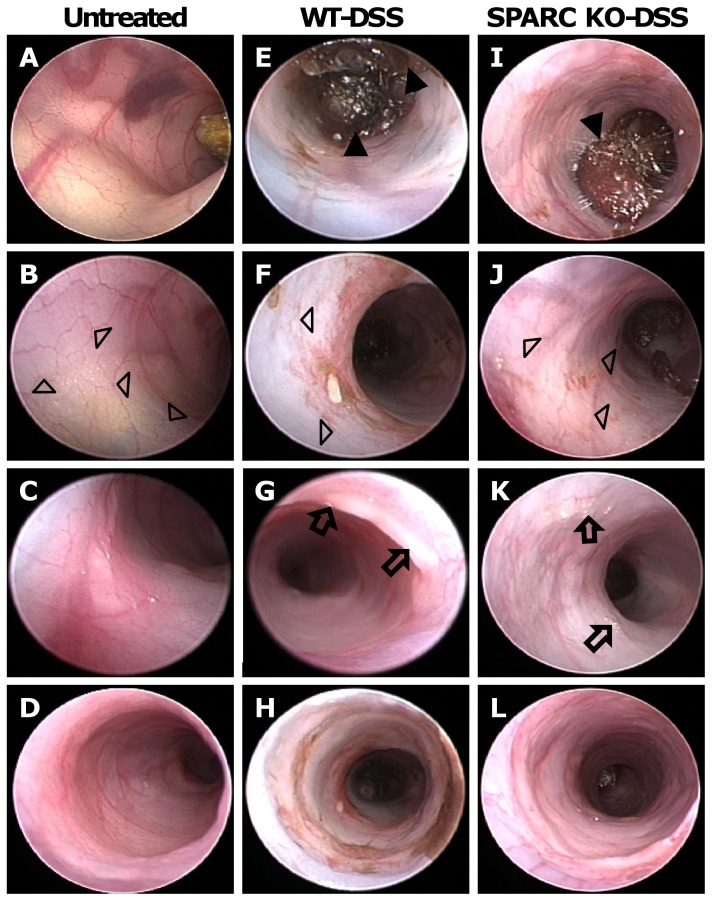
Micro-endoscopic images of WT and SPARC KO mice after DSS treatment. Representative endoscopic images at day 6 showing the colon of healthy control (A, B, C, D), DSS-treated WT (E, F, G, H) and DSS-treated SPARC KO (I, J, K, L) mice. When treated with DSS for 7 days, both WT and SPARC KO mice showed signs of inflammation: loose and bloody stool (solid triangle in E, I), thickening of colonic wall with reduction in translucency (F, J), altered vascular pattern **(**open triangle in F, J) and present of fibrin (arrow in G, K). The lowest panel (D, H, L) showed the colon closest to rectum, which has a different fibrosturcture to the distal and proximal colon.

Endoscopically, untreated control mice had solid stool, clearly visible vascularity with translucency of the intestinal wall (***Figure 2*** ***A, B, C***). By contrast, there was endoscopic inflammation that was maximal in the distal 2–3 cms of the colon, in all the DSS-treated animals. On day 6, using the MEICS, both the WT and SPARC KO mice demonstrated marked endoscopic signs of mucosal inflammation with soft, poorly formed and bloody stools (solid triangle in ***Figure 2*** ***E, I***), mucosa thickening (***Figure 2*** ***F, J***), spontaneous bleeding, loss of visible blood vessel structures (open triangle in ***Figure 2*** ***F, J***), and the presence of fibrin (arrow in ***Figure 2*** ***G, K***). All these features were noted to be worse in the WT DSS-treated mice indicating a greater level of inflammation. Of note was the greater level of bleeding, mucosal thickening and changes in the vascular pattern in the DSS-treated WT animals (***Figure 2*** ***E, F, G)*** compared to the SPARC KO mice (***Figure 2*** ***I, J, K***).

**Figure 3:**
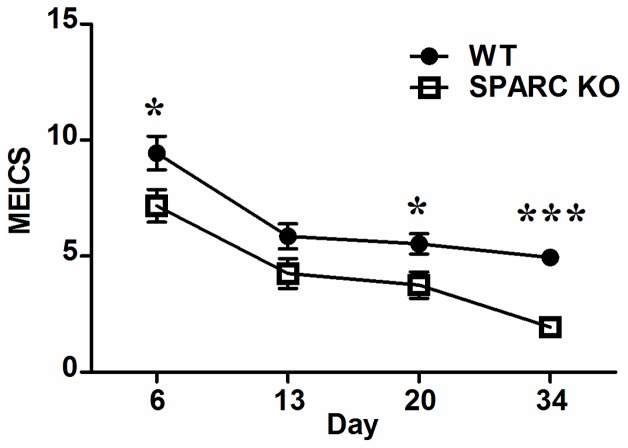
MEICS of WT and SPARC KO mice. Line graph comparing SPARC KO-DSS to WT-DSS mice at difference time points. Values are mean ± SEM based on records of 8–15 mice per time point from 3 different experiments (*p<0.05, ***p<0.001).

While the untreated controls all had a score MEICS of 0, the WT animals had higher MEICS scores than SPARC KO mice at days 6 (p = 0.039), 14 (p = 0.067), 20 (p = 0.019) and 34 (p<0.0001) (***Figure 3***). After DSS removal, the stool reverted to normal and the colonic wall translucency and vascular pattern gradually returned to normal by day 13 and remained endoscopically normal at days 20 and 34. Granularity of the mucosa surface consistent with ongoing inflammation, however, was observed in the distal colon at day 34 in the WT animals (Image not shown).

**Figure 4:**
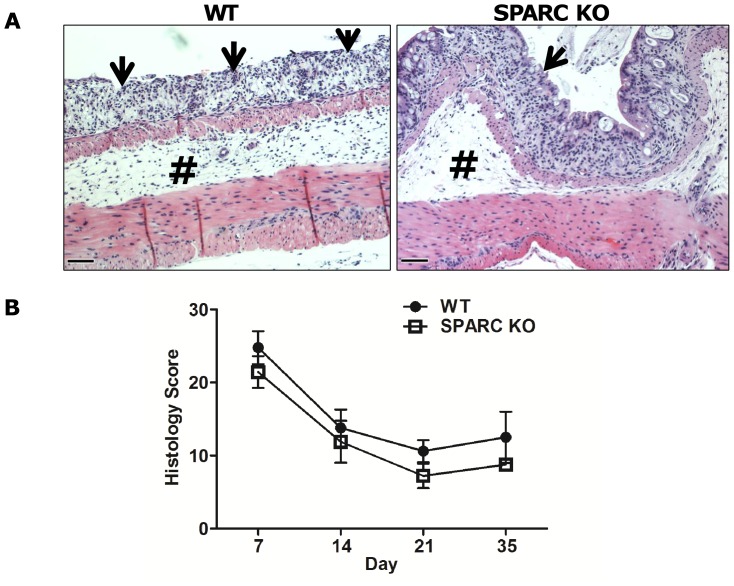
Histological assessment of SPARC KO and WT colons. **A)** Representative photographs of H&E-stained sections of the distal colon at day 7 demonstrating ulceration, mononuclear cell infiltrate involving part to all of the colonic wall with disruption of the normal crypt architecture and loss of goblet cells (slim arrow) and submucoa oedema (**#**). Bar: 100 µm. B) Extent of inflammation quantified and expressed as an inflammation score. Each time point consisted of 10–15 mice from 3 different experiments. Value is mean ± SEM. (*p<0.05)

The untreated WT and SPARC KO controls all had a histological score of 0, while the DSS-treated SPARC KO animals had a lower histological inflammatory score (***Figure 4B***) than the WT mice but this did not reach statistical significance. At day 7, moderate to severe inflammation was seen in both WT and SPARC KO mice, with transmural inflammation particularly seen in the WT animals. There was colonic crypt lost with a reduction in the goblet cell numbers, focal ulceration inflammatory cell infiltration (slim arrow in ***Figure 4A***) and oedema of the submucosa (# in ***Figure 4A***). Between days 14 to 35, regeneration was identified by re-epithelisation and proliferation of new crypts. DSS-treated WT mice, however, still demonstrated mucosal crypt damage and ulceration at day 21 in contrast to the mucosal reconstitution observed in the SPARC KO mice (Image not shown). By day 35, based on the inflammation severity and regeneration subscores as described by Dieleman *et al* [18], all the SPARC KO DSS-treated mice (n = 13) had completely resolved the colonic mucosal inflammation, while 50% (7/14) of WT DSS-treated animals still demonstrated mucosal inflammation (χ^2^ (1) = 8.78, p<0.01).

### Colonic cytokine production

**Figure 5:**
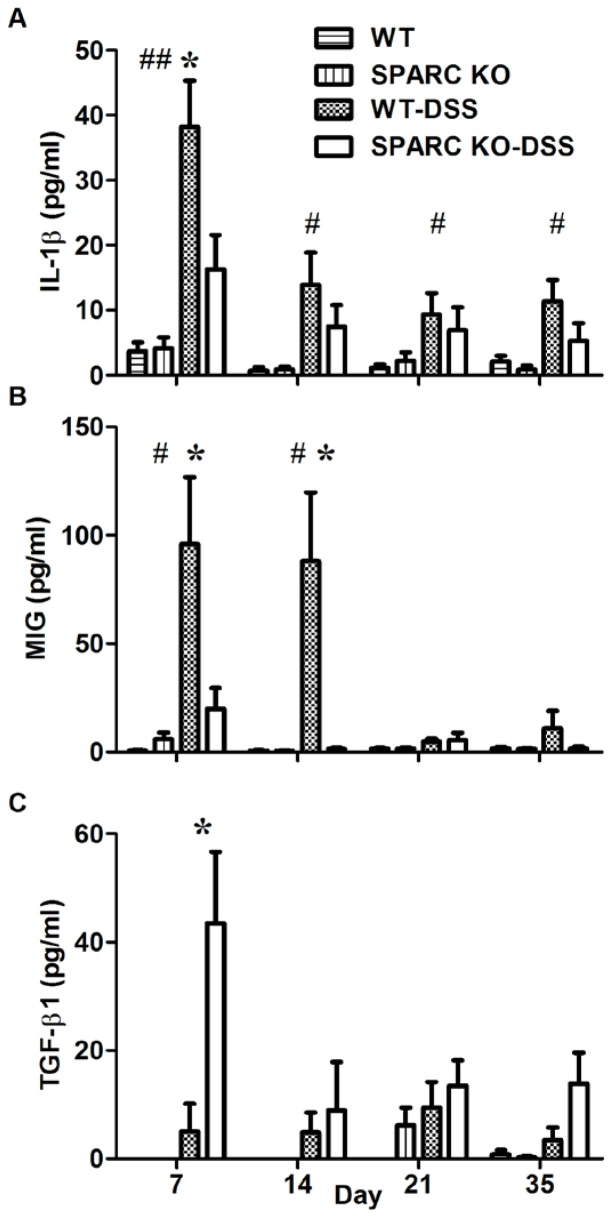
IL-1β, MIG and TGF-β1 production in the colon of WT and SPARC KO mice measured with CBA and ELISA. Mean ± SEM, *p = 0.05 when comparing WT-DSS mice to SPARC KO-DSS mice; # p = 0.05, ##p<0.01 when comparing untreated WT mice to WT-DSS mice (*n*  = 10–20 mice per group from 3 different experiments per time point).

No differences were observed between the untreated WT and SPARC KO control mice for the pro-inflammatory cytokines IL-1β, IL-6 or TNF-α at any time point and had less cytokine secretion than all the DSS-treated animals at all time points (p<0.05). The DSS-treated animals demonstrated significantly elevated levels of IL-1β (p<0.01; ***Figure 5A***), IL6 (p = 0.03, Data not shown) and TNF-α (p = 0.029; Data not shown) at day 7 compared to untreated controls. The levels fell rapidly following DSS withdrawal. WT DSS-treated animals demonstrated higher levels of IL-1β, IL6 and TNF-α compared to the SPARC KO mice at all time points and these correlated with the MEICS and histological scores. Only IL-1β secretion (***Figure 5A***) at day 7, however, was significantly higher in WT DSS-treated animals compared to the DSS-treated SPARC KO mice (p = 0.025).

As chemokines are critical elements in regulating cell trafficking to inflammatory sites [19] these were also measured. MIG (monokines induced by IFN-γ) was detected in the untreated animals and was significantly induced following DSS treatment at 7 days in WT animals (p = 0.011, ***Figure 5B***) and also in SPARC KO animals but to a lesser extent. MIG protein levels were significantly higher in DSS-treated WT mice (***Figure 5B***) compared to DSS-treated SPARC KO mice at day 7 (p = 0.031) and 14 (p = 0.014).

TGF-β1 proteins levels were also significantly higher in DSS-treated SPARC KO compared to WT animals at day 7 (p = 0.017; ***Figure 5C***). There were no significant differences in the secretion patterns observed between the WT and SPARC KO animals after DSS treatment for the Th-1 (IFN- γ), Th-2 (IL-4, IL-5, IL-13), and Th-17 (IL-17A, IL-12/IL23p40) related cytokine levels. No significant trend was observed for the anti-inflammatory IL-10 cytokine and other chemokines (RANTES, MCP-1, MIP-1α, MIP-1β) measured in this study as well.

### Treg cells levels

**Figure 6:**
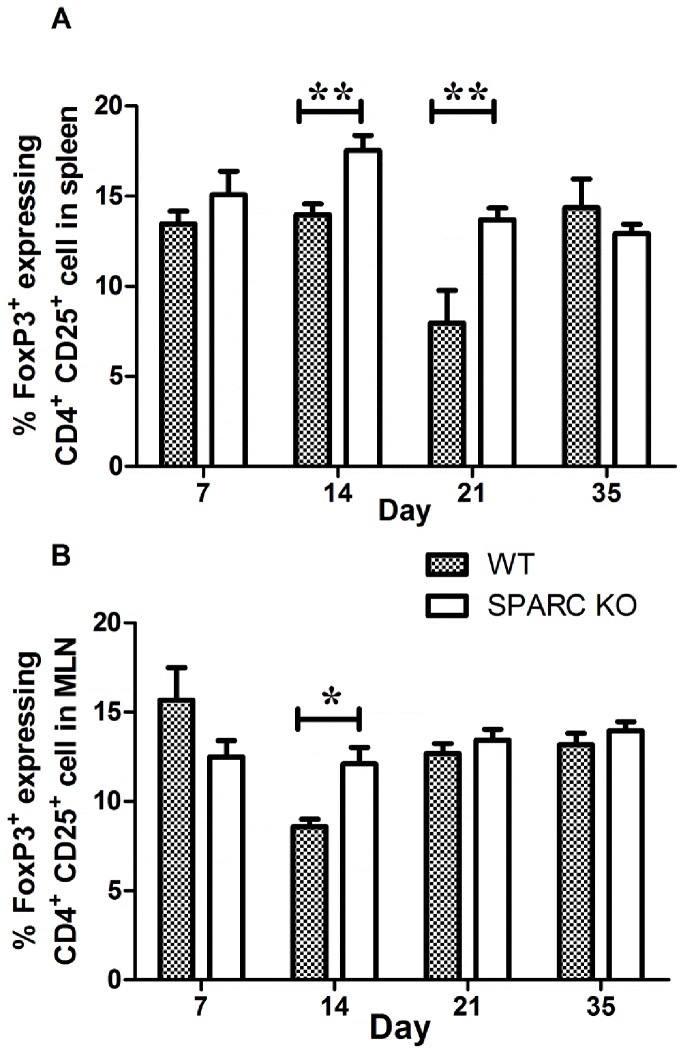
Percentage of Treg cells (CD4+CD25+FoxP3+) in the spleen (A) and MLN (B) from SPARC KO and WT mice. Cells were isolated from spleen and MLN on day 7, 14, 21, and 35 following DSS treatment and determined by flow cytometry. Data represent the mean percentage of Treg cells ± SEM from 3 independent experiments each consisting of 4-5 animals; *p<0.05; **p<0.01.

The percentage of Treg cells (CD4+CD25+FoxP3) was higher in the spleen (13.2% vs 15.8%; p<0.01) and MLNs (8.55% vs 11.14%; p = 0.013) of untreated SPARC KO compared to WT mice (Data not shown). Following DSS-treatment Treg cell percentages were also increased in the SPARC KO mice above that of the WT animals at day 14 in both the spleen (p<0.01) and MLN (p<0.01); percentages were also increase at day 21 in the spleen (p<0.01) (***Figure 6***).

**Figure 7:**
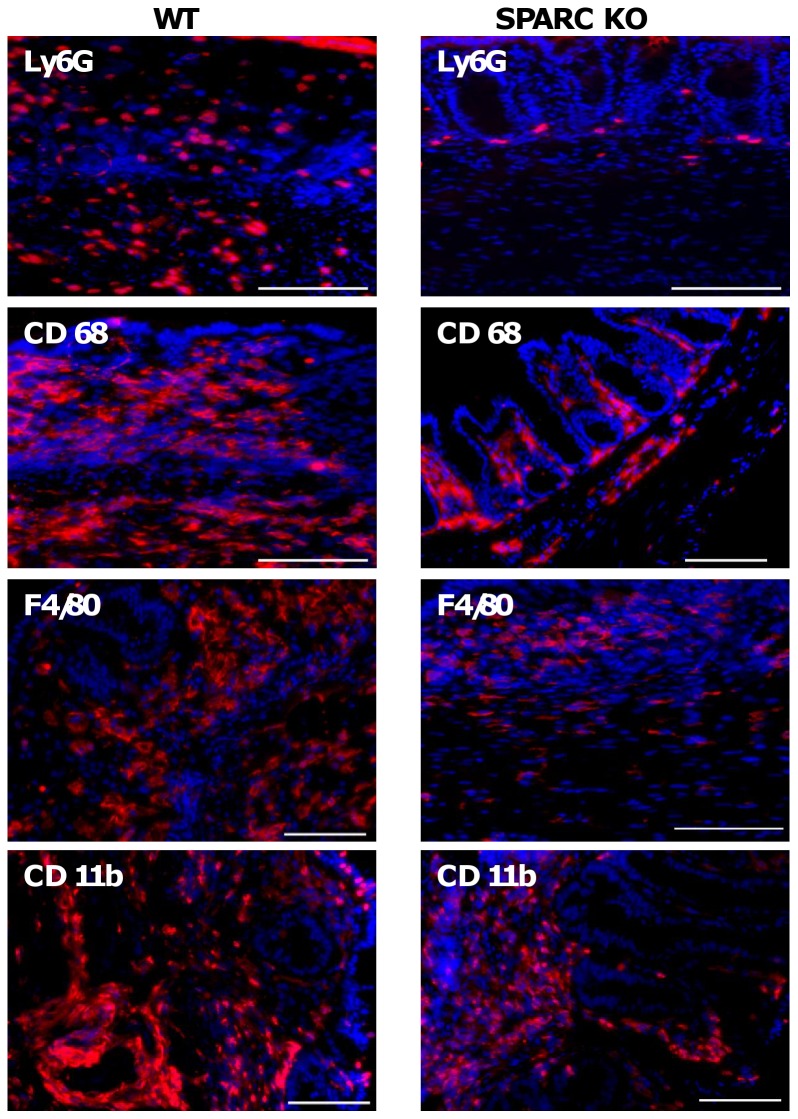
Ly6G+, CD68 + F4/80+ and CD 11b+ cellular levels in WT and SPARC KO colons after DSS treatment. Representative images of in situ visualisation of colonic neutrophils and macrophages. Positive staining (red) and co-stained nuclei (blue). Bar: 100 µmm

**Innate cell pattern in WT and SPARC KO Mice.** In control animals, consistent with the absence of inflammation, there were almost no neutrophils present and those that were present were located within the lamina propria (LP). Following DSS treatment in both groups, greater numbers of neutrophils were observed in the LP and submucosa, particularly at sites of ulceration (***Figure 7***). Ly6G+ cells (a neutrophil marker) were significantly more abundant in DSS-treated WT animals than in SPARC KO mice at day 21 (1.69 fold, p<0.001) and 35 (9.93 fold, p<0.001) consistent with more inflammation.

In control mice, CD68+ cells (a macrophage marker) were located primarily in the LP, with low numbers also detected in the submucosa. Following DSS treatment, CD68+ cells increased with greater cell numbers located within the submucosa (***Figure 7***). WT animals had statistically greater numbers of CD68+ cells than SPARC KO mice following DSS treatment at day 14, (1.36 fold, p<0.001), 21 (1.45 fold, p<0.001) and 35 (1.50 fold, p<0.001) again consistent with a greater level of inflammation. When stained with another macrophage marker, F4/80, a similar trend was observed. At day 14 (1.07 fold, p<0.001) and 21 (1.69 fold, p<0.001), F4/80+ cells were observed to be greater in the WT-DSS mice in both LP and submucosa when compared to the DSS-treated SPARC KO mice (***Figure 7***; Day 7 and 35 time points were not investigated). CD11b was also assessed and this stains for macrophages, monocytes and neutrophils (***Figure 7***). More CD11b+ cells were observed in the DSS-treated WT compared to the DSS-treated KO mice at day 21 (1.09 fold, p = 0.02) and 35 (1.18 fold, p<0.001) in line with greater levels of inflammation in the WT animals.

## Discussion

SPARC can play an active role in cell migration, tissue regulation and wound healing [6], [20]-[22]. A role for SPARC in the colon, however, has not been studied and the literature suggests that the role of SPARC in inflammation could be both organ and model specific [9], [10], [23]. As the DSS-induced colitis model exhibits inflammatory and histopathological features that are similar to human colitis, it can be used as a model to assess the effect of the presence of SPARC and its role in the colonic inflammation of IBD [2], [18], [24], [25].

Ingestion of DSS for 7–10 days induces acute inflammation [1], [4], [5], [26]. Chronic inflammation can also be achieved by the administration of 3–5 cycles of DSS with a DSS-free period between each cycle [1]. A single DSS cycle, however, is sufficient to induce chronicity in the C57BL/6 mice and was used in this study [5]. The data presented, however, demonstrated a potential pro-inflammatory role for SPARC in the DSS-induced model of murine colitis. The clinical parameters of colitis were all noticeably less in the SPARC KO mice and these were accompanied by significantly lower micro-endoscopic inflammatory scores. Of note, WT mice had a tendency to develop a chronic, slow healing inflammation when all of the SPARC KO mice had fully resolved the intestinal inflammation by the end of the study.

Endoscopic examination of the colon is important for the diagnosis and assessment of colonic diseases. The use of the mouse micro-endoscopic system provided valuable and accurate information in the assessment and monitoring of the intestinal inflammation and its resolution [17] and it clearly demonstrated less inflammation in the KO animals. The endoscopic scoring also appears to be a more sensitive method of evaluating inflammation levels than either histology, or the use of surrogate markers used to evaluation disease activity such as weight loss and stool consistency.

At day 7, both, WT and SPARC KO DSS-treated animals, showed similar levels of histological inflammation, but the SPARC KO mice had less severe inflammation at each subsequent time point. This suggests that the SPARC KO animals may be able to heal more rapidly. The regeneration sub-score alone, however, did not detect any difference suggesting that the histology scoring of colonic regeneration is difficult and not particularly sensitive. Regeneration, however, can only be assessed following the peak of inflammation (day 7) but histological assessment was only possible at weekly intervals. This raises the possibility that the point of peak regeneration may have been missed in the SPARC KO animals. One solution to this limitation may be the inclusion of more time points over a shorter time interval, this, however, was not feasible in this study.

SPARC effects on wound healing appear to vary depending on the tissue type, but the findings in this study are similar to those of Bradshaw and colleagues where accelerated cutaneous wound healing was observed in SPARC-null mice [27]. The anti-proliferative properties of SPARC [6] may also be a potential cause of a slower healing in the WT animals as re-epithelialisation principally begins with proliferation of the neighbouring epithelium [18]. If indeed SPARC KO animals heal faster than their WT littermates then this could also be related to the inhibition by SPARC of other important mediators in healing like platelet derived growth factor, vascular endothelial growth factor and basic fibroblast growth factor. In addition, the dermal structure of SPARC KO mice has been shown to be different to WT animals with smaller collagen fibrils suggesting an alteration in collagen bundling in the absence of SPARC. These findings were support the concept that a less structured ECM would allow for a faster cellular migration resulting in faster healing [20], [28].

Cytokines and chemokines interact in a complex and integrated way and are central to the pathogenesis of IBD and this study evaluated a wide range of cytokines and chemokines [29], [30]. It is known that SPARC can enhance macrophage, neutrophil and epithelial cell migration [31], [32] and it is primarily these cells that secrete the pro-inflammatory cytokines IL-6, IL-1β and TNF-α [5]. IL-1β is secreted by macrophages and its activation within the mucosa and sub-mucosa [33], [34] induces and perpetuates human colitis [35]. The tissue damage observed in WT animals would thus be enhanced by the greater levels of the pro-inflammatory cytokines observed in this study. Macrophages are also thought to be the major source of MIG and their presence within the intestinal mucosa may be able to shift the cytokine profile from a predominance of TNF-α/IL-1 to IFN-γ and subsequently to MIG [36]. MIG has recently been shown to play a role in mediating T cell recruitment to site of inflammation in the heart tissue [37] and its expression is elevated in mixed cellularity subtype of Hodgkin’s lymphoma patients where this disease is thought to be similar to the inflammatory phase observed in wound healing process [38]. With lower chemokine levels, migration of leucocytes, especially neutrophils, into the colon of the SPARC KO animals would be reduced. A similar effect can also be observed in chemokine receptor-deficient mice, which are protected from the DSS-induced inflammation due to less infiltration by immune cells [29], [39].

The SPARC KO animals were observed to have higher colonic TGF-β1 levels, which contrasted with the report by Socha and colleagues that found lower TGF-β expression in the kidney in the absence of SPARC [9]. It is unclear why more TGF-β was observed in SPARC KO colons, but it may be secondary to the unique immune adaptations of the colon which is colonised with great numbers of Treg cells that are able to modulate the immunosuppressive activity of TGF-β. In addition, the intestinal mucosa promotes epithelial restitution after injury through the increased production of bioactive TGF-β1 by the epithelial cells [40] and subepithelial myofibroblasts [41] and thus the increase in TGF-β1 may contribute to the maintenance of intestinal barrier integrity and intestinal healing in SPARC KO mice resulting in less inflammation.

In summary, this study demonstrates that SPARC is associated with increased inflammation in the DSS-induced model of colonic inflammation, which seems to be associated with a longer time to tissue healing. This is most likely secondary to SPARC’s effect on cell migration and ECM regulation, which subsequently modulate the inflammatory cell infiltration and the secretion of pro-inflammatory cytokines.
